# Commentary: Eye acupuncture therapy for insomnia: asystematic review and network meta-analysis

**DOI:** 10.3389/fneur.2026.1776033

**Published:** 2026-05-19

**Authors:** Qianqian Li, Xiaohan Wang, Guosheng Zheng, Yunfeng Xia, Ying Hai

**Affiliations:** 1 Department of Acupuncture and Moxibustion, Liaoning University of Traditional Chinese Medicine, Shenyang, China; 2 Department of Acupuncture and Moxibustion, Affiliated Hospital of Liaoning University of Traditional Chinese Medicine, Shenyang, China

**Keywords:** biological characteristics of the eye, eye acupuncture, functional mechanisms of eye acupuncture for insomnia, insomnia, structural basis of eye acupuncture for insomnia

We have carefully read the article entitled Eye acupuncture therapy for insomnia: A Systematic Review and Network Meta-Analysis by Shi et al., published in *Frontiers in Neuroscience* in December 2025 (1), and been greatly inspired by their rigorous work. Through a comprehensive systematic review and network meta-analysis, their study conclusively confirmed the significant advantages of eye acupuncture, either as a monotherapy or in combination with other interventions (including pharmaceutical and non-pharmaceutical approaches), in improving clinical efficacy, reducing Pittsburgh Sleep Quality Index (PSQI) scores and ameliorating Traditional Chinese Medicine (TCM) syndrome scores in patients with insomnia. Notably, their finding that eye acupuncture combined with body acupuncture and eye acupuncture combined with wrist-ankle acupuncture are particularly effective therapeutic strategies provides valuable evidence-based support for the clinical application of eye acupuncture in the management of insomnia.

As researchers with long-standing focus on the mechanistic study of eye acupuncture, we propose that the discussion on the therapeutic efficacy of eye acupuncture for insomnia in their study can be further deepened by integrating the biological characteristics of the eye. Eye acupuncture is a specialized acupuncture therapy that treats systemic diseases by needling acupoints in the periorbital region. Its therapeutic effects can be elucidated from the perspectives of ocular embryonic development, structural foundations (anatomical and histological characteristics) and functional regulation (multi-system modulatory effects) (2), thus forming a complete mechanistic framework where structural bases underpin functional effects and functional effects rely on structural foundations.

## 1 Developmental basis of eye acupuncture for insomnia

**Figure 1:**
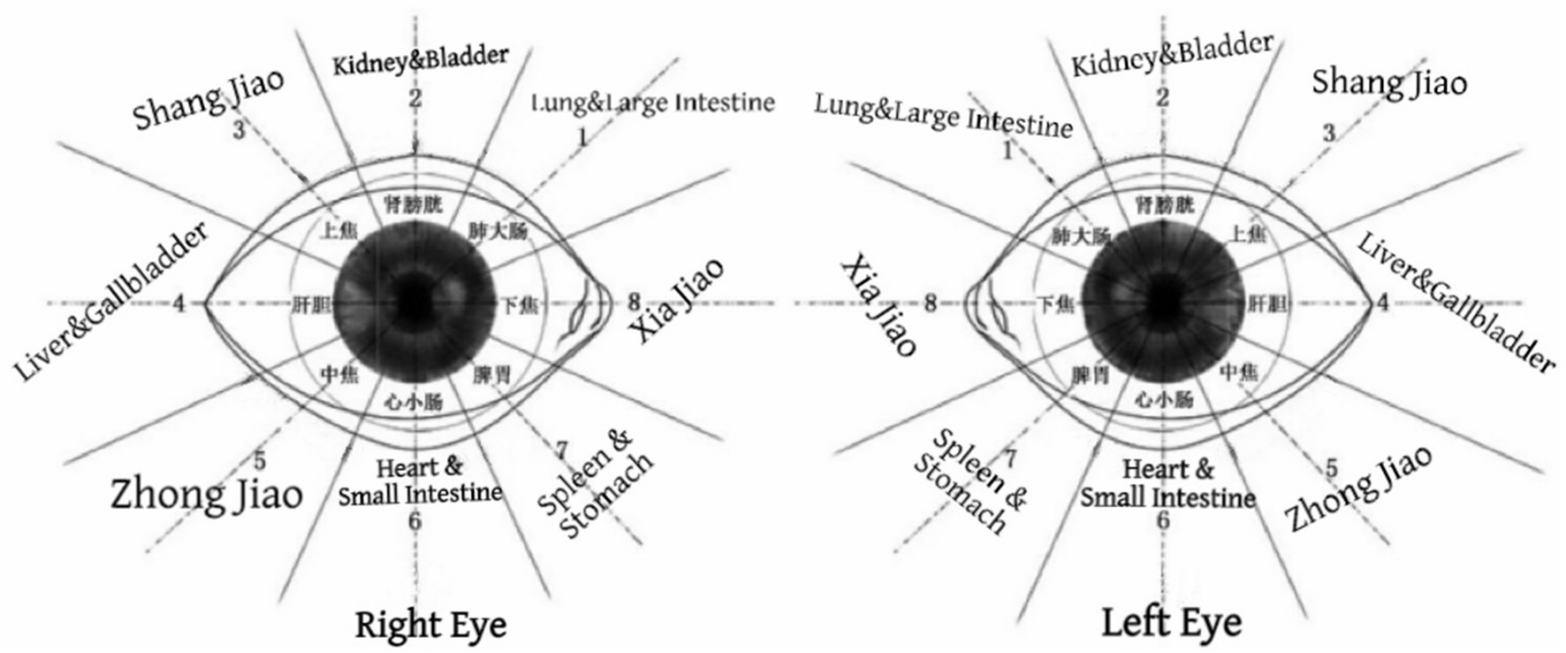
Eye acupuncture region map. Region 1: lung and large intestine; Region 2: kidney and bladder; Region 3: upper Jiao; Region 4: liver and gallbladder; Region 5: middle Jiao; Region 6: heart and small intestine; Region 7: spleen and stomach; Region 8: lower Jiao.

The eye primordium develops from the combined differentiation of the ectoderm and mesoderm. The mesoderm differentiates into blood vessels, visceral tissues, meridians, and mesenchymal tissues, which is highly consistent with the concept of the “Sanjiao (Triple Energizer)” in traditional Chinese medicine (TCM). TCM regards Sanjiao as a functional communication system that runs through the human body, circulates qi, blood, and body fluids, and connects the zang-fu organs and the whole body. Modern embryology has confirmed that blood vessels, visceral interstitium, connective tissue, fascia, and mesenchymal tissues all originate from the mesoderm. These structures collectively form a continuous connective tissue network throughout the body, which is highly consistent with the core characteristics of Sanjiao: “promoting the flow of all kinds of qi, transporting water and fluids, and connecting the exterior and interior” (3). Meanwhile, studies have explicitly proposed that the substantial structure of Sanjiao corresponds to the connective tissue and mesenchymal system. The core components of Sanjiao, including membrane-origin (moyuan), interstices (couli), fascia, tendons, and bones, are mainly composed of connective tissue. Connective tissue exhibits continuity, conductivity, and body fluid transport functions, constituting the modern material basis for the “channeling” effect of Sanjiao. Furthermore, mesodermal differentiation fully matches the functions of Sanjiao: the vascular system circulates blood and qi, mesenchymal and connective tissue pathways mediate signal conduction, and visceral supporting structures connect the zang-fu organs. Such structures and functions are completely consistent with the core connotation of Sanjiao in TCM: “promoting the flow of primordial qi, transporting water and fluids, and nourishing the whole body.” The connective tissue network derived from the mesoderm serves as the structural basis connecting the eyes with the zang-fu organs and meridians of the whole body, and also provides embryological evidence for the TCM theory of “correspondence between the eye and zang-fu organs.” Therefore, eye acupuncture by stimulating periorbital acupoint regions corresponding to specific zang-fu organs can rapidly regulate systemic qi, blood circulation, and zang-fu functions through meridian conduction. For instance, in insomnia caused by disharmony between the heart and kidney, stimulating acupoints in the heart and kidney regions calms the mind and soothes the spirit; in insomnia due to liver-spleen disharmony, regulating acupoints in the liver and spleen regions relieves liver stagnation and invigorates the spleen. This fundamentally corrects the zang-fu imbalance underlying insomnia, thus explaining the therapeutic efficacy of eye acupuncture in the treatment of insomnia. As shown in Figure 1.

## 2 Structural basis of eye acupuncture for insomnia

Like other acupuncture therapies, eye acupuncture exerts remote therapeutic effects through mechanical stimulation of acupoint regions, a form of physical stimulation. Its unique acupuncture effects are realized by virtue of the distinctive anatomical structure and specific tissue distribution in the periorbital region, which constitutes the material basis that distinguishes eye acupuncture from other acupuncture modalities and underpins its therapeutic effects on insomnia. The structural mechanisms of eye acupuncture are centrally associated with the high-density connective tissue, specific cellular distribution and biomechanical characteristics of the extraocular muscles in the periorbital region, all of which are oriented toward the effective reception and conduction of mechanical stimulation induced by acupuncture needling.

### 2.1 Mechanical conduction potential of extraocular muscles and periorbital high-density connective tissue

Extraocular muscles are rich in high-density connective tissue and exhibit prominent viscoelasticity and mechanical conduction properties. Studies have demonstrated that the adhesion torque between extraocular muscles and the sclera increases with the elevation of eye movement velocity and the angle of interface separation. At a high eye movement velocity (approximately 461 deg/s), the adhesion torque accounts for 0.50%−0.53% of the total torque. Although this proportion is relatively small, its mechanical behavior has been verified by contact and pull-out tests as well as finite element simulation, suggesting a potential role in local mechanical transmission and the capacity to effectively receive the physical stress generated by acupuncture needling (4). Periorbital connective tissues, such as the orbital fascia and the attachment sites of extraocular muscles, are the primary targets of eye acupuncture needling, and their structural characteristics support their role as key mediators of mechanical signal transmission. These regions are abundant in the extracellular matrix (ECM), which not only serves as a structural scaffold but also acts as a crucial medium for mechanotransduction. Accumulating evidence has indicated that cells interact with the ECM through membrane-bound adhesion proteins (e.g., integrins) to sense and respond to external mechanical stimulation, thereby mediating the mechanotransduction process and regulating cellular processes such as cell migration and tissue morphogenesis (5). This mechanism is likely to play a pivotal role in the response of periorbital connective tissue to the physical stimulation of acupuncture. In addition, the integration of the ECM and the cytoskeleton is essential for mechanical perception. For example, in a dynamic tissue-like model constructed with human tendon fibroblasts, the deposition and mechanical integration of the ECM were found to significantly enhance cellular sensitivity to stretch and reduce the strain threshold required to trigger calcium signaling (6). Similar mechanisms may exist in extraocular muscles and their attachment sites, enabling the subtle physical stress induced by acupuncture needling to be effectively perceived and converted into intracellular signals. Notably, the ECM not only provides structural support but also modulates cellular functions through its dynamic mechanical properties. For instance, a fiber hydrogel complex regulated by near-infrared light can reversibly alter its mechanical properties to modulate the activity of mechanosensitive ion channels (e.g., Piezo1 and TRPV4), thereby promoting angiogenesis (7). This finding suggests that changes in the physical state of the ECM itself can act as a signal source to influence cellular behavior. Therefore, during eye acupuncture intervention, the local tissue deformation caused by needling may activate relevant mechanosensitive signaling pathways by altering the mechanical microenvironment of the ECM, thereby initiating subsequent biological responses.

In summary, extraocular muscles and their surrounding connective tissues possess the structural and functional foundations for receiving and conducting the physical stress of acupuncture, owing to their high-density ECM, prominent viscoelasticity and dynamic adhesion characteristics with the sclera. As the core mediator of mechanical signal transmission, the ECM can convert physical stimulation into biochemical signals recognizable by cells through integrin-mediated mechanotransduction pathways, providing a potential mechanistic basis for the functional effects of eye acupuncture therapy.

### 2.2 Specific cell distribution in periorbital acupoint areas: the core initiating role of mast cells (MCs)

Among the various cell types in acupoint-related tissues, mast cells are widely recognized in the academic community as the key switch of acupuncture-specific sensory receptors. Mast cells are predominantly distributed around blood vessels and peripheral nerves, form close interactions with the nervous and vascular systems, and can regulate the neuroendocrine-immune (NEI) network, thus being referred to as the “mobile targets” of acupuncture (8). A study investigating the distribution pattern of cutaneous mast cells in different human body regions identified high-density mast cell aggregation zones around peripheral nerve trunks and somatic orifices. A comparison with acupoint maps revealed that, except for the trunk, the distribution characteristics of mast cells are highly consistent with the arrangement of acupoints along the fourteen meridians. Relevant mapping studies have also shown that all micro-acupuncture systems (e.g., auricular, scalp, hand, foot, ocular, facial, and umbilical acupuncture) are constructed with high-density mast cell zones as the core. This conclusion confirms that the density distribution of cutaneous mast cells is highly correlated with acupoints and micro-acupuncture systems, providing histological empirical evidence for the neuroimmunological mechanisms underlying acupuncture effects (9). In addition, numerous studies have demonstrated that subcutaneous mast cells are highly enriched at acupuncture acupoints and express a variety of transient receptor potential vanilloid (TRPV) channels, including TRPV1, TRPV2, and TRPV4 (10, 11). These channels are classified as mechanosensitive ion channels and can respond to local mechanical stimulation transmitted by collagen fiber deformation induced by acupuncture (9). During acupuncture needling, mechanical force is transmitted to the mast cell membrane through the dense collagen network, activating TRPV channels on the cell surface and triggering calcium influx, which in turn induces mast cell degranulation (10). Following degranulation, mast cells release a variety of bioactive mediators, including hyaluronic acid (HA), histamine, 5-hydroxytryptamine (5-HT), adenosine triphosphate (ATP) and adenosine (10). Among these mediators, HA is not only a structural polysaccharide but also exhibits regulatory effects on inflammation and tissue remodeling (12). Previous studies (13) have also conducted preliminary explorations on MCs in the periorbital acupoint areas of healthy rats and found that the number of MCs aggregated in the skin tissue of periorbital acupoint areas of rats is significantly higher than that in non-acupoint areas, and acupuncture can induce the degranulation of MCs in acupoint areas to release bioactive substances such as HA, which may be one of the mechanisms by which eye acupuncture exerts its therapeutic effect. In the periorbital region, acupuncture-induced mast cell degranulation and the subsequent release of HA and histamine are considered the core steps in converting local mechanical stimulation into biochemical signals (9). These released mediators can act on receptors of peripheral sensory nerve endings (e.g., histamine H1 receptors and adenosine A1 receptors), reduce the electrical activity of primary sensory neurons, and thereby produce an analgesic effect (10). In addition, signal molecules released by mast cells can also participate in local and even systemic functional regulation by modulating vascular permeability, recruiting immune cells and mediating neuro-immune interactions (14).

In summary, mast cells sense the mechanical signals transmitted by collagen fibers during acupuncture needling through TRPV1/2/4 channels on their cell membranes, trigger degranulation and release a variety of mediators (including HA and histamine). This process not only completes the conversion of local mechanical signals into biological signals in the periorbital region but also forms an important bridge connecting local acupuncture stimulation with systemic physiological regulation.

### 2.3 Mechanosensitive characteristics of periorbital fibroblasts

A large number of fibroblasts are present in the periorbital connective tissue, and these cells act as key mechanosensitive target cells and intermediate cells in the process of eye acupuncture stimulation. Periorbital fibroblasts can directly receive the mechanical stress transmitted by the ECM through acupuncture manipulation and become activated upon stimulation, thereby initiating a series of biological responses. Specifically, activated fibroblasts can release alarmin proteins such as interleukin-33 (IL-33), participate in the regulation of local macrophage activity, and further mediate immune regulation and tissue repair processes (15). In addition, these activated fibroblasts can also initiate the release of local cytokines and trigger immune regulatory responses, converting physical mechanical stimulation into functional biological effects—an important intermediate link for the therapeutic effects of eye acupuncture therapy (8, 16). Further studies have demonstrated that orbital fibroblasts express functional mechanosensitive Piezo1 channels, and the *in vitro* activation of these channels can inhibit their adipogenic differentiation, suggesting that mechanical stress can regulate the biological behavior of orbital fibroblasts through the Piezo1 pathway (17). This finding provides molecular support for the mechanistic effects of mechanical stimulation induced by eye acupuncture. Meanwhile, in a three-dimensional culture environment, fibroblasts have been shown to exhibit direction-dependent mechanical sensitivity and achieve mechanotransduction through calcium signal conduction (18), indicating their high responsiveness to the mechanical signals exerted by acupuncture needling.

In summary, fibroblasts in the periorbital connective tissue are not only structural support cells but also key functional cells responsible for sensing and transducing acupuncture-induced mechanical signals. Through the cytokine release and immune regulatory responses triggered upon activation, these cells convert the physical stimulation of eye acupuncture into local and even systemic therapeutic effects.

## 3 Functional mechanisms of eye acupuncture for insomnia

The mechanical signals generated by eye acupuncture through stimulating the periorbital structure are converted into biological signals locally and then transmitted to the central nervous system (CNS) and the whole body through the NEI network. Ultimately, eye acupuncture ameliorates insomnia through multi-dimensional regulation of core pathological processes, including neurotransmitter imbalances, neuroinflammation and endocrine rhythm disorders. Meanwhile, the regulation of local cellular functions in the periorbital region provides a preliminary functional basis for systemic regulation, forming an overall functional pathway of local regulation → signal transmission → central integration → sleep improvement.

### 3.1 Initiation of biological signals triggered by periorbital structural stimulation

The mechanical stimulation induced by eye acupuncture first triggers cellular-level functional regulation in the periorbital region, laying a foundation for subsequent systemic regulation and serving as the initial link of its functional effects. Periorbital mast cells are located in the vicinity of nerve endings and undergo degranulation under the mechanical stimulation of eye acupuncture, releasing a variety of bioactive mediators including HA (8). This process has multiple physiological implications. First, it enhances neural signal conduction: the released HA can specifically activate the branches of the trigeminal and vagus nerves, thereby significantly improving the afferent efficiency of acupuncture signals to the CNS. This neural activation not only increases the perceptual intensity of peripheral stimulation but also provides an input basis for the subsequent central regulation of the sleep-wake cycle. Second, it inhibits local inflammatory responses: the downregulation of proinflammatory factor release associated with mast cell degranulation helps maintain local immune homeostasis in the periorbital region and prevents excessive inflammation from interfering with the normal conduction of neural signals. This anti-inflammatory effect may indirectly modulate the central neuroinflammatory state by regulating the cytokine profile in the local periorbital microenvironment. Third, it regulates the neural microenvironment to support the balance of sleep-related neurotransmitters: mediators released by mast cells can alter the chemical composition of the microenvironment surrounding local nerves, thereby affecting the synthesis, release and reuptake of neurotransmitters (e.g., 5-HT and GABA), and providing support for the CNS to maintain a normal sleep-wake cycle. In addition, the anatomical distribution characteristics of periorbital mast cells also provide a structural basis for their role as signal initiation nodes. Studies have demonstrated that choroidal mast cells are densely distributed around the optic nerve, with distribution differences among different mouse strains (19), suggesting that their specific localization facilitates the rapid reception of mechanical stimulation and subsequent response. This spatial arrangement may enable eye acupuncture stimulation to activate mast cells more efficiently, thereby initiating subsequent signal cascades.

Periorbital fibroblasts are also mechanosensitive and become activated under eye acupuncture intervention, participating in the remodeling of the local immune-neural microenvironment. Activated fibroblasts can secrete a variety of cytokines such as TGF-β, IL-1β and IL-6 (20). These cytokines participate in local tissue repair and immune regulation on the one hand, and transmit signals to the CNS through the NEI network on the other hand. Among these cytokines, TGF-β exhibits prominent anti-inflammatory and immunosuppressive effects, while IL-1β and IL-6 participate in neuroplasticity and circadian rhythm regulation at physiological levels. By dynamically regulating the release of these cytokines, fibroblasts help maintain the homeostasis of the periorbital microenvironment and prevent excessive inflammation from interfering with neural signal conduction (19). Meanwhile, studies have shown that periorbital fibroblasts and mast cells act synergistically under mechanical stimulation to jointly regulate the local immune microenvironment, and may further affect the central inflammatory state and neuronal function through the NEI network (21). For example, in a cutaneous inflammation model, the interaction between fibroblasts and mast cells can regulate leukocyte migration and collagen deposition, suggesting that a similar synergistic mechanism may exist in periorbital tissues.

In summary, ocular acupuncture stimulation activates periorbital mast cells and fibroblasts, which release HA and a variety of cytokines, respectively, forming a biological signal initiation platform in the periorbital region with the functions of neural signal amplification, anti-inflammatory regulation and microenvironment homeostasis maintenance. This local regulation not only lays a foundation for the subsequent transmission of signals to the CNS through the NEI network but also provides a preliminary functional basis for systemic sleep improvement.

### 3.2 Multi-target synergistic regulation of the sleep-wake rhythm

After being transmitted to the CNS through the NEI network, the local signals of ocular acupuncture integrate with the general mechanistic pathways of acupuncture for insomnia, and realize sleep regulation by targeting three core pathological processes: neurotransmitter imbalances, neuroinflammation and endocrine axis dysfunction. These three regulatory targets cooperate with and mutually support each other, constituting the core functional mechanisms of ocular acupuncture in the treatment of insomnia.

#### 3.2.1 Regulating neurotransmitter balance and restoring the dynamic homeostasis of the sleep-wake cycle

In a PCPA-induced insomnia mouse model, probiotic intervention rebalances the neurotransmitter network by enhancing 5-HT synthesis, inhibiting its degradation, and simultaneously reducing the levels of dopamine (DA) and norepinephrine (NE) (22); modified Suanzaoren Decoction has also been shown to increase central 5-HT levels, reduce DA and NE levels, and ameliorate the glutamate (Glu)/γ-aminobutyric acid (GABA) imbalance (23). In addition, during non-rapid eye movement (NREM) sleep, neuromodulators (e.g., 5-HT, NE and acetylcholine) exhibit synchronous rhythmic fluctuations. Pharmacological or optogenetic intervention of any system can affect the release of other modulators, suggesting a high degree of synergy of the multi-transmitter system (24). Eye acupuncture may optimize the ratio of inhibitory (e.g., GABA) and excitatory (e.g., Glu) transmitters through a similar mechanism, enhance central inhibitory function, prolong deep slow-wave sleep, and inhibit the overactivity of the arousal system, thereby alleviating sleep onset insomnia and sleep maintenance disorders.

#### 3.2.2 Inhibiting neuroinflammatory responses and remodeling brain functional connectivity

Clinical studies have demonstrated that regional global brain connectivity in the globus pallidus and prefrontal cortex is reduced in patients with chronic insomnia following acupuncture intervention, and this alteration is significantly correlated with decreased serum IL-6 levels. Gene enrichment analysis has further revealed that astrocyte activation and neuroinflammation-related signaling pathways are implicated in this process (25). Animal experiments have also provided empirical support for this mechanism. Studies have found that electroacupuncture suppresses the NF-κB pathway by upregulating OTULIN expression, which in turn reduces the levels of TNF-α, IL-1β and IL-6 and inhibits glial cell activation (26); additionally, bone marrow mesenchymal stem cell (BMSC) intervention alleviates neuroinflammation and modulates the gut microbiota by inhibiting the AMPK/NF-κB pathway and glial cell activation (24). These lines of evidence indicate that eye acupuncture may transmit anti-inflammatory signals mediated by periorbital fibroblasts and mast cells to the central nervous system (CNS) via the neuroendocrine-immune (NEI) network, remodel aberrant brain functional connectivity, and restore homeostasis within the sleep regulatory network.

#### 3.2.3 Regulating HPA axis function and restoring neuroendocrine rhythm

Patients with chronic insomnia typically exhibit hyperactivation of the hypothalamic-pituitary-adrenal (HPA) axis, which is characterized by elevated cortisol levels (27). Eye acupuncture exerts a distinct regulatory effect on this pathological state: it downregulates abnormally elevated levels of hypothalamic corticotropin-releasing hormone (CRH), pituitary adrenocorticotropic hormone (ACTH) and adrenal cortisol (CORT), repairs the negative feedback function of the HPA axis, normalizes its circadian rhythm, and thereby alleviates the persistent state of systemic hyperarousal (28). This regulatory mechanism has been validated in numerous studies. Electroacupuncture reduces CRH, ACTH, and CORT levels by inhibiting the Nesfatin-1/ERK/CREB pathway in the paraventricular nucleus of the hypothalamus, which mitigates anxiety-like behaviors and HPA axis hyperactivation (29); Ultrafine powder of Dendrobium officinale also ameliorates neuroendocrine disorders in a subhealthy state by downregulating CRH, ACTH, and cortisol levels (30), Moreover, chronic sleep restriction itself can induce HPA axis rhythmic disturbances, which is manifested as reduced basal corticosterone levels accompanied by an enhanced stress response, with notable gender-specific differences (31). The regulatory effect of eye acupuncture on the HPA axis is not an isolated phenomenon but forms a synergistic regulatory network with neurotransmitter balance and neuroinflammation inhibition. For instance, HPA axis activation is often accompanied by decreased central 5-HT levels, elevated DA/NE levels and excessive release of proinflammatory factors, and eye acupuncture can modulate these three systems synchronously to collectively promote the restoration of the normal sleep-wake cycle.

**Figure 2:**
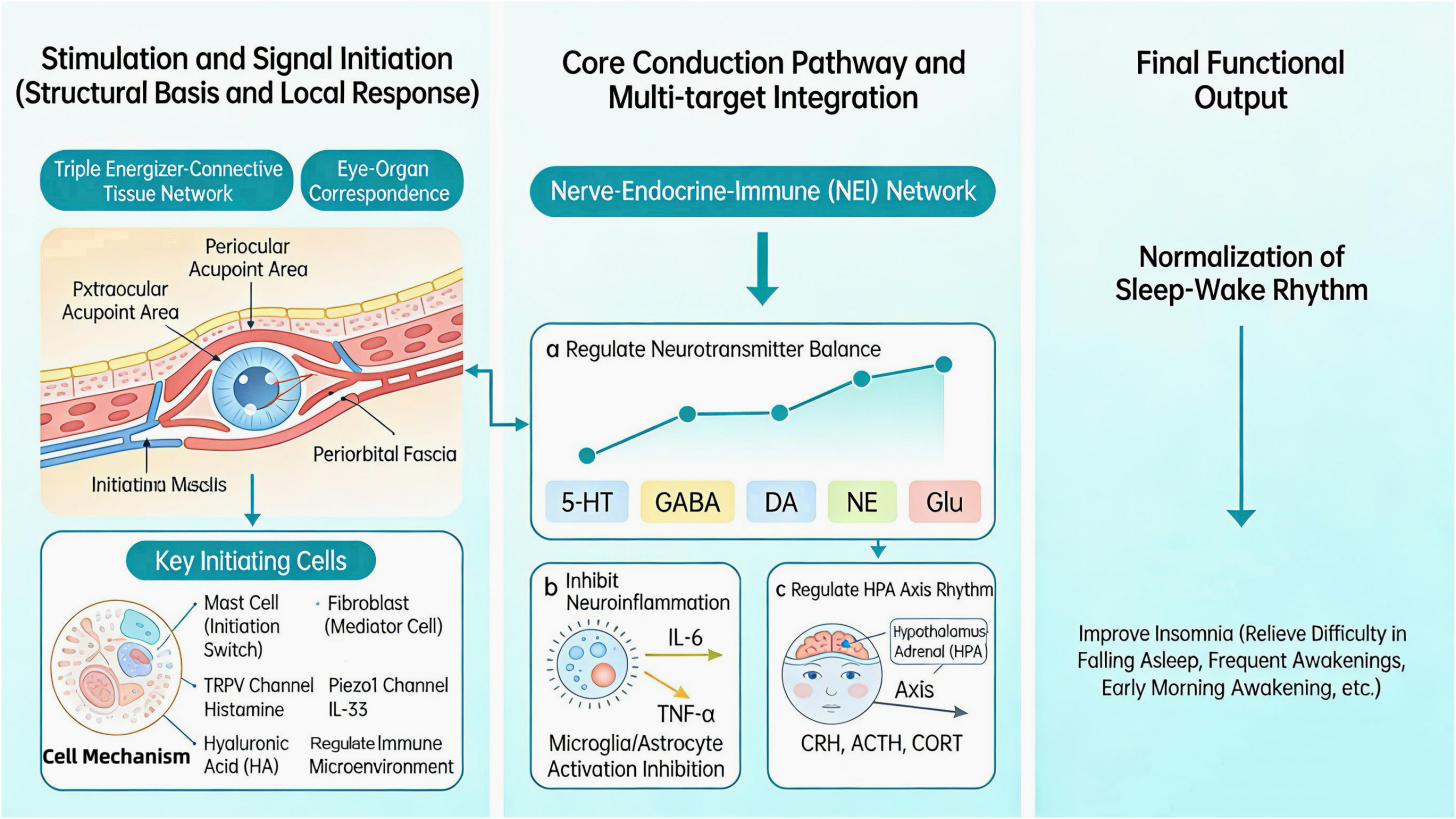
Mechanism of eye acupuncture in the treatment of insomnia.

In summary, eye acupuncture integrates local stimulatory signals via the NEI network and exerts synergistic regulatory effects on central neurotransmitter balance, neuroinflammatory status and HPA axis function at the CNS level. These three regulatory dimensions mutually support and reinforce one another, constituting the core multi-target mechanism underlying the therapeutic efficacy of eye acupuncture for insomnia. As shown in Figure 2.

Although direct empirical evidence elucidating the underlying mechanisms of eye acupuncture for insomnia remains relatively scarce, the unique developmental, structural, and functional characteristics of the eye undoubtedly underpin the rapid onset and remarkable therapeutic efficacy of this therapy. These mechanisms effectively complement the findings of your esteemed study, furnishing a more robust theoretical basis for the clinical application of eye acupuncture in insomnia management and subsequent mechanistic investigations in this field—for which additional single-factor animal experiments and cell-based assays focusing specifically on eye acupuncture are warranted.
